# A Mechanistic Model for the Extraction of Phenolics from Grapes During Red Wine Fermentation

**DOI:** 10.3390/molecules24071275

**Published:** 2019-04-02

**Authors:** Konrad V. Miller, Roberto Noguera, Jordan Beaver, Cristina Medina-Plaza, Anita Oberholster, David E. Block

**Affiliations:** 1 Department of Chemical Engineering, University of California, One Shields Avenue, Davis, CA 95616, USA; vonmiller@ucdavis.edu (K.V.M.); rcnoguera@ucdavis.edu (R.N.); 2 Department of Viticulture and Enology, University of California, One Shields Avenue, Davis, CA 95616, USA; jwbeaver@ucdavis.edu (J.B.); cmedinaplaza@ucdavis.edu (C.M.-P.); aoberholster@ucdavis.edu (A.O.)

**Keywords:** wine, phenolic extraction, kinetic model, grape seed structure

## Abstract

Phenolic extraction is a critical part of red wine making. Though empirical models of phenolic extraction kinetics exist, the current level of mechanistic understanding does not allow for accurate predictions. In this work, we propose a mechanistic model for the extraction of phenolics from grape skins and seeds as a function of temperature and ethanol. This model examines the release of phenolics, the adsorption of phenolics onto grape material, and the disappearance of anthocyanins from solution. Additionally, we performed epifluorescence microscopy to explore our finding that seed tannins’ release rate appears independent of concentration, and found that the grape seed appears to ablate over fermentation. We also determined the activation energy of anthocyanin disappearance, in good agreement with similar systems. The proposed model results in an excellent fit, and increases the understanding of phenolic extraction and the ability to predict and optimize product outcome in red wine making.

## 1. Introduction

Phenolic extraction is of critical importance to red winemaking. Color (anthocyanins) and mouthfeel (tannins) are extracted from grape skins and seeds over the course of fermentation [1]. While this phenomenon has been studied in great depth, the exact mechanisms of phenolic extraction during fermentation are still largely unknown. Past studies into the kinetics of grape phenolic extraction during winemaking can be broken into three clusters: qualitative explorations, empirical data fitting, and purely theoretical models.

First, qualitative studies of extraction rate seek to explore the gross impact of winemaking conditions, such as temperature, cap (grape solids that aggregate during fermentation) management, and fermentation progression. Lerno [2] found that phenolic extraction rate and final phenolic profile are strong functions of temperature, with the fraction of seed-derived tannins increasing with temperature. However, final phenolic extraction was found to be a weak function of cap management, as determined by Lerno [3]. Finally, Lerno [4] found that extraction of phenolics from grape solids was found to slow down between mixing events in the fermenter and then speed up after mixing, indicating a diffusion driven process. These studies are critical to develop a mechanistic understanding of phenolic extraction, but do not allow for quantitative predictions or simple extrapolation to larger industrial systems.

Second, totally empirical models of phenolic extraction have been explored in literature with predictive power. For example, Singleton and Draper [5] measured the extraction of seed phenolics as a function of ethanol and temperature and proposed a logarithmic fit. Boulton [1] proposes several empirical kinetic models for the extraction of phenolics from grapes. Setford [6] offers an excellent review of the current state of the art in empirical modeling of the extraction and reaction of phenolics in wine. Similar work has been performed by Bucic-Kojic [7] in the industrial solvent extraction of polyphenols from grape seeds, but this work is not directly applicable to winemaking practices. These fits are accurate and have good predictive power, though they do not consider temperature or ethanol concentrations. The empirical nature of these models makes it difficult to extend them to practical winemaking fermentations.

First principle models of phenolic extraction from grapes in literature is limited. An excellent example of a first principles description of grape phenolic extraction is the anthocyanin extraction model proposed by Setford [8], which models the unsteady-state leaching of anthocyanins from grape solids in wine making conditions. This work rigorously accounts for mass transfer of anthocyanins, but does not account for other key phenolics present (i.e., tannins), the adsorption of phenolics onto grape solids, and the reaction of phenolics in solution. In non-grape plant extraction systems, Simeonov [9] performed analyses of transient diffusion for the extraction of tobacco concrete from tobacco leaves, with good agreement between simulation and experiment. Chilev [10] did extensive work in describing extraction from *Cotnius coggygria* for various solvent systems and developed a method for the estimation of diffusion coefficients through plant material; however, this system is substantially simpler (well mixed temperature with a constant solvent) than a red wine fermenter. 

**Figure 1:**
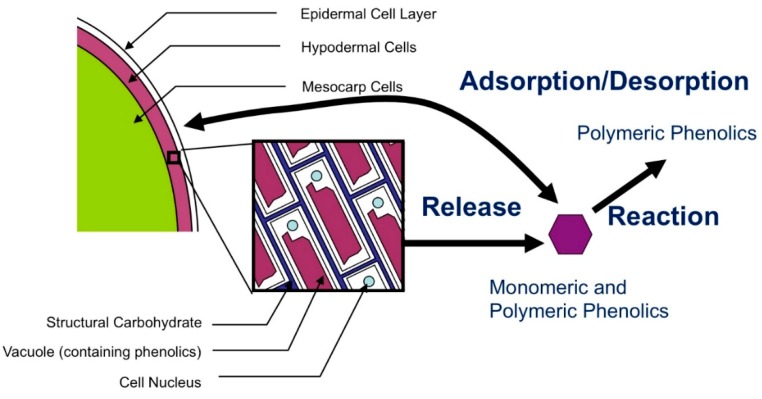
Diagram of processes involved in phenolic extraction. Phenolics are released from grape cells into solution, where they are free to either enter adsorption equilibrium onto the surface of the grape cell walls, or react (i.e., polymerization, degradation) in solution.

There is an opportunity to improve on the current understanding of phenolic extraction by deriving a mechanistic model, where the form of the model is based in first principles and the specific constants are numerically derived where sufficient data do not yet exist. This paper attempts to address this opportunity. The model derived incorporates the effect of temperature and ethanol concentration, and accounts for (i) the release of phenolics from skin and seed cells, (ii) the adsorption/desorption of phenolics onto grape material, and (iii) the apparent disappearance of phenolics due to reactions, such as polymerization or degradation. These phenomena are summarized in Figure 1. The model is derived and validated using previously reported data [2]. 

## 2. Results

### 2.1. Model Development

In order to create an effective mechanistic model, we must identify the underlying phenomena and create mathematical descriptions for each of them. In this case, the relevant phenomena are the release of phenolics from grape cells, the adsorption equilibrium of phenolics onto the surface of grape cells, and the reaction of phenolics (Figure 1). This extraction model makes several simplifying assumptions to allow for a direct kinetic analysis of extraction data, as delineated throughout this Model Development section. Grapes are segregated into two phenolic reservoirs: skins, which contain both anthocyanins and tannins, and seeds, which contain tannins. An overall material balance on phenolic species “*i*” (anthocyanins or tannins) is then applied by equating the initial amount of *i* in the grape ($C_{i,G}^{0}$) with the current available but unreleased in grape ($C_{i,G}$), plus amount adsorbed on surface ($C_{i,S}$), plus amount free in solution ($C_{i,F}$), plus amount reacted away ($C_{i,R}$), plus the amount unavailable for release ($C_{i,U}$) in the form of Equation (1):

(1) $$C_{i,G}^{0}=C_{i,G}+C_{i,S}+C_{i,F}+C_{i,R}+C_{i,U}$$

The amount of extractable phenolics ($C_{i,G}^{0}-C_{i,U}$) increases with temperature, as seen in Lerno [2].

Next, it is assumed that the rate of release of phenolics (from grape skin or seeds to liquid) follows a power law form, where release rate is equal to a constant (*k_G_*) multiplied by the driving force for release (concentration of unreleased phenolics minus concentration free in solution) to some arbitrary power (α), in the form of:

(2) $$\frac{dC_{i,G}}{dt}=-k_{G}*{\left(C_{i,G}-C_{i,F}\right)}^{\alpha }$$

We then assume the rate of release and reaction of phenolics is much slower than adsorption/desorption of phenolics onto grape cell wall material, i.e., the system is in adsorption equilibrium. We can express the competitive adsorption of anthocyanins and tannins onto grape cell wall material via a competitive Langmuir model, as indicated in Equation (3):

(3) $$q_{i}\equiv \frac{grams\;i\;ads}{grams\;adsorbate}\equiv \frac{C_{i,S}}{\Gamma \times \varphi }=\frac{{\left(q_{i}\right)}_{m}K_{i}C_{i,F}}{1+\sum_{j}K_{j}C_{j,F}}$$

For this adsorption model, Γ is taken to be the amount of grape cell wall material per gram grape, φ is the fraction of cell wall material available for adsorption, q_i,m_ is the adsorption capacity of species *i* on cell wall material, and K_i_ is the Langmuir adsorption equilibrium constant of species *i*. While Γ and φ are both free parameters in the model, φ is fixed at 20%, as Battista [11] observed that only ~20% of grape skin cells are exposed to the surface. Beaver [12] determined Langmuir adsorption data fits (K, adsorption equilibrium constant, and *q*, the adsorption capacity) for tannins on grape cell wall material as a function of temperature and ethanol concentration, with Medina-Plaza [13] doing the same for anthocyanins. This is the only source of the model’s dependence on ethanol concentration. There parameters, and all others in the model, are functions of temperature.

The rate of phenolic disappearance (as degradation or reaction) is also modeled as a power law form:

(4) $$\frac{dC_{i,R}}{dt}=-k_{R}\times C_{i,F}{}^{\beta }$$

where k_R_ is the reaction rate constant, and β is the reaction rate order. Multiple sources have observed anthocyanins disappearing from solution on the order of days to weeks, while overall tannin concentration does not appear to decrease in this time span [2,4]. In this work, we limit the analysis of phenolic disappearance (be it to degradation, polymerization, etc.) to the apparent disappearance of anthocyanins.

### 2.2. Estimating Release and Reaction Model Parameters

These parameters have not been reported in literature. As such, we calculated them by fitting data from previously published work [2]. Parameters derived for phenolic release and reaction are assumed to be functions of temperature. Adsorption kinetics, as reported by Beaver [12] and Medina-Plaza [13], are taken to be functions of both temperature and ethanol, and are not a result of fitting in this work.

First, we used the concentration versus time data of Lerno [2] to explore the release kinetics over a range of temperatures, since these data also reported the absolute magnitude of phenolics available for extraction. As elucidated in the Materials and Methods section, the rise in skin phenolic concentrations at early time points is directly proportional to the difference between the amounts yet unreleased and the amount in solution, indicating a first-order dependence on the concentration driving force, as expected from a diffusion-driven process (α = 1 for skin phenolics). The rate of seed phenolic release was constant with time (zero order in unreleased tannin concentration, α = 1 for seed phenolics), which matches prior observations [4]. Similar analysis was performed at later time points to determine the rate of disappearance of species from solution (zero order, β = 0).

**Figure 2:**
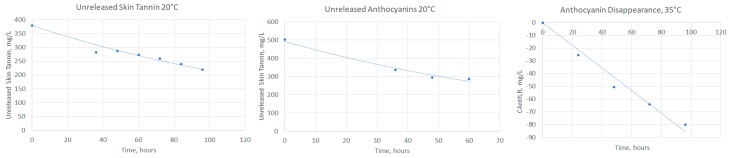
Kinetic fits for extraction phenomena. (**a**) Release of skin tannins at 20 °C with an integrated first order fit (y = 379.4 × e^−0.006x^). (**b**) Release of anthocyanins at 20 °C with an integrated first order fit (y = 492.5 × e^−0.01x^). (**c**) Anthocyanin disappearance at 35 °C with a zero-order fit (y = −0.89x).

As an example, Figure 2 shows the fitting of release phenolics for skin tannins at 20 °C, Figure 2b the release skin anthocyanins at 20 °C, and Figure 2c the disappearance of anthocyanins at 35 °C. The difference between current and peak liquid phenolic concentrations are plotted for early time points, with the fitted exponential decay constant equal to the time constant. The good agreement indicates that the rate of release is proportional to the driving force, as expected.

The final derived form of the phenolic extraction equations are as follows:

(5) $$C_{i,G}^{0}=C_{i,G}+C_{i,S}+C_{i,F}+C_{i,R}+C_{i,U}$$

(6) $$\frac{dC_{Anth,skin}}{dt}=-k_{G,Anth}\times \left(C_{Anth,G}-C_{Anth,F}\right)$$

(7) $$\frac{dC_{Tan,skin}}{dt}=-k_{G,Tan,Skin}\times \left(C_{Tan,G}-C_{Tan,F}\right)$$

(8) $$\frac{dC_{Tan,seed}}{dt}=-k_{G,Tan,Seed}$$

(9) $$q_{i}\equiv \frac{grams\;i\;ads}{grams\;adsorbate}\equiv \frac{C_{i,S}}{\Gamma \times \varphi }=\frac{{\left(q_{i}\right)}_{m}K_{i}C_{i,F}}{1+\sum_{j}K_{j}C_{j,F}}$$

(10) $$\frac{dC_{Anth,R}}{dt}=-k_{R,Anth}$$

### 2.3. Examining the Temperature Dependence of Release and Reaction Parameters

**Figure 3:**
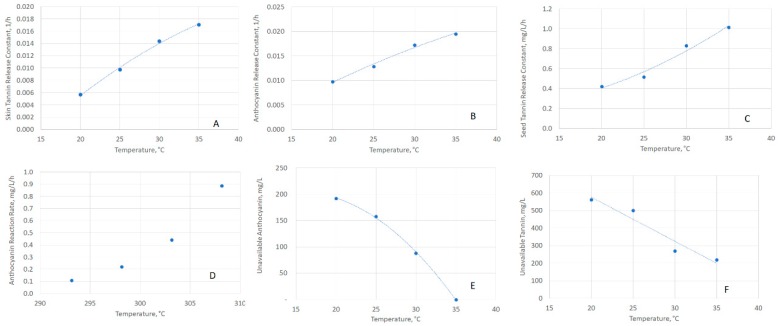
Extraction parameters as a function of temperature. (**A**) Skin-Tannin Release Constant, (**B**) Anthocyanin Release Constant, (**C**) Seed-Tannin Release Constant, (**D**) Anthocyanin Reaction Rate Constant, (**E**) Anthocyanin Unavailable, (**F**) Skin-Tannin Unavailable.

With the kinetic constants calculated, it is important to understand the temperature dependence of each. Figure 3 plots (with regressions) the derived values as a function of temperature for the skin tannin release constant (Figure 3a), skin anthocyanin release constant (Figure 3b), seed tannin release constant (Figure 3c), anthocyanin reaction rate constant (Figure 3d, regression omitted due to Arrhenius fit), unavailable anthocyanin concentration (Figure 3e), and unavailable skin tannin concentration (Figure 3f). Arrhenius parameters were derived for the disappearance rate constant for anthocyanins, with a pre-exponential constant of 1.38 × 10^18^, mg/L/h and an activation energy of 107.3 kJ/mol. This value is in agreement with literature [14,15,16,17], as seen in Table 1. Arbitrary functions were used to explain the temperature dependence for all parameters, except for anthocyanin disappearance, as seen in Table 2.

### 2.4. Evaluating Model Fit to Experimental Data

**Figure 4:**
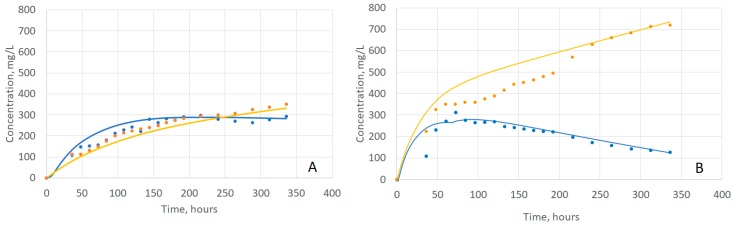
Phenolic Extraction Model Fits. The temperature extremes of 20 °C (**A**) and 35 °C (**B**) are shown to indicate the range of fits. Blue dots: anthocyanin data. Blue curve: model anthocyanin fit. Orange dots: tannin data. Orange curve: tannin fits.

Given the model developed and the estimated parameters, we wanted to evaluate the ability of the model to describe the overall extraction process. Figure 4A,B show the fitting results at 20 °C and 35 °C, respectively. Overall, this model has an excellent qualitative and quantitative fit with Lerno [2] data. Table 3 lists the R^2^ value for the anthocyanins and tannin fits at each temperature.

### 2.5. Physical Basis for Observed Form of Skin and Seed Release Expressions

The expression derived for skin tannin and anthocyanin release is first order in concentration, as expected. The expression derived for seed tannin release rate is zero order in concentration, an unexpected result. To further investigate phenolic release from skins and seeds over the course of fermentation, we examined grape skin exteriors and seed cross sections under epifluorescence microscopy.

**Figure 5:**
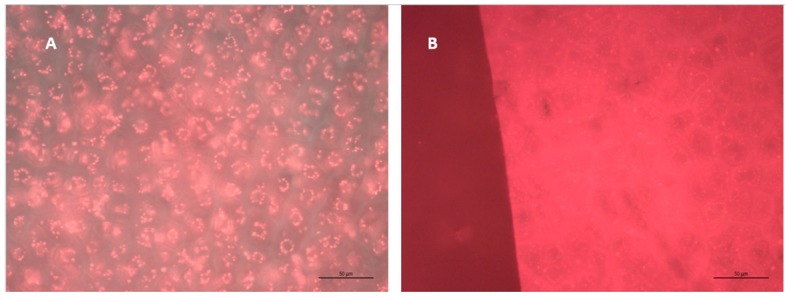
Epifluorescence microscopy of the exterior of grape skins at 400× magnification. Note the fluorescing bright dots in the 5–10 micron range, and the disappearance of fluorescing dots over time. (**A**) Day 0 of fermentation, (**B**) Day 14 of fermentation.

While the anthocyanin release data shows a clear first order dependence, as expected, the physical mechanism of release is still unknown. We observed bright fluorescing circular structures in the skin cells (Figure 5) under epifluorescence microscopy with an excitation of 426–446 nm and emission filter from 465–495 nm. These structures became dimmer and sparser over time, which corresponds with the measured release of anthocyanins into liquid. This is consistent with Drabent [18], who observed fluorescence in red cabbage anthocyanins with excitation at ~410 nm and emission at ~500 nm. The fluorescent dots we observed had diameters in the 5–10 micron range. Okamoto [19] observed anthocyanoplasts (anthocyanin rich vacuoles) in grape skin cells in the 3–30 micron range, while Kolb [20] observed anthocyanin structures in grape skin cells via epifluorescence microscopy in the 5–7 micron range, although they did not identify these structures as anthocyanoplasts. As such, we believe these bright fluorescent dots to be anthocyanoplasts in the skins.

Data fitting shows the release of phenolics from the seeds to be consistent with zero order kinetics. One possible explanation we wanted to explore was that the apparent zero order release of phenolics was the result of multiple competing phenomena. Therefore, we examined seed cross sections to try and plumb the mechanism of seed release.

**Figure 6:**
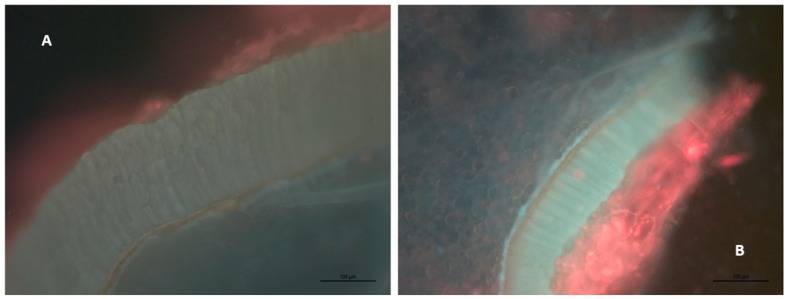
Epifluorescence microscopy of the outside of grape seed sections at 200× magnification. Note the presence of residual pulp (red material) and the columnar structure of the outer waxy layer (endocarp). The outer layer appears to ablate over time. (**A**) Day 0, (**B**) Day 14.

**Figure 7:**
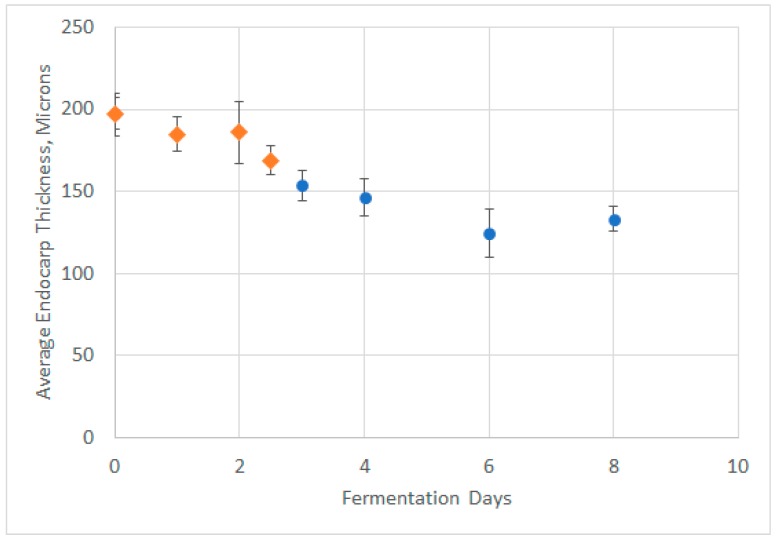
Seed Endocarp thickness over time. Note that two time points from repeat fermentation with the same grapes are overlaid: 0–2.5 days, orange diamonds; 0–8 days, blue circles (note first points overlap). The outer layer noticeably shrinks over time, resulting in a shorter diffusion path for seed tannins over the course of fermentations.

The lamella of the seed outer waxy layer is highly visible under the SAQ filter. Some residual berry flesh was also visible on the outside of the endocarp. Figure 6A,B (at 200×) show seed endocarp prior to fermentation and at the end of extraction, with the layer between the seed endosperm and the pulp obviously thinner. Therefore, we measured the thickness of the lamella in the outer layer over time in two separate experiments (Figure 7), and observed a statistically significant trend towards decreasing endocarp thickness over the course of fermentation.

## 3. Discussion

Overall there is excellent agreement between the experimental data from Lerno [2] and this new model, especially considering that major parts of the model (i.e., adsorption equilibria) are not fitted from the concentration data used to derive the kinetics. A successful model will be a useful tool not only in understanding the physical phenomena, but in optimizing phenolic extraction in real red wine fermentations.

It is interesting to note that the rate of skin phenolics (both anthocyanins and skin tannins) release appears to be first order with respect to concentration driving force, which is expected for a diffusion driven process. Phenolic release rate increases with temperature, which matches a priori expectations. The amount of accessible phenolics for extraction also increases with temperature, another intuitive finding. Also of interest is that anthocyanins, which are substantially smaller molecules than tannins, have a larger release constant at lower temperatures, as expected in a diffusion driven process.

A more surprising conclusion is that the seed tannins release rate has a zero-order dependence on seed tannin concentration. This has been observed in prior work [4]. Mass transfer rates would be expected to be first order in concentration, not zero order. Our results from Figure 6 and Figure 7, which indicate that the seed outer layer shrinks over the course of fermentation, may suggest an explanation for this unusual behavior. The expression for flux from Fickian diffusion is:

(11) $$J_{i}=\frac{D_{i}}{L}\Delta C_{i}$$

where *J_i_* is the diffusive flux, *D_i_* is the diffusion coefficient, *L* is the path length for diffusion, and Δ*C_i_* is the concentration difference along the path length. It could be the case that as Δ*C_i_* (concentration driving force between seed and liquid tannin concentration) decreases, *L* also decreases due to ablation of seed endocarp, keeping flux constant. This would explain the discrepancy and is consistent with the analysis performed by Simeonov [9] on the impact of path length on diffusive extraction of plant material. This also matches observations from Thorngate [21], which found that while phenolics are present in the “outer soft coat” of grape skins in much higher abundance than endosperm, the majority of tannins and procyanidins are present in the “hard seed coat”, the brown hull between the outer soft coat and the endorsperm. This is consistent with our findings above—over time, the outer soft coat ablates, which reduces the mass transfer resistance to the release of tannins from deeper in the seed, resulting in a constant rate of seed tannin release.

We derived a zero-order rate law for the disappearance of anthocyanins in solution. While there is no such thing as a true zero order reaction rate law, apparent zero order rate laws can be observed when a reactant (in this case, anthocyanins) is present in abundance compared to either another reactant, or when a catalyst (i.e., ionic copper, enzymes, etc.) is sufficiently diluted as to be always saturated with reactants. Rhim [15] also observed a zero-order loss of pigmented colors in grape juice, with a very similar activation energy (92.8 kJ/mol) to that determined in this work (107.3 kJ/mol). The activation energy determined for anthocyanin disappearance matches those found in literature for fruit anthocyanin degradation well, as seen in Table 1. This agreement suggests that some level of confidence can be placed in the results. 

Even with the excellent agreement between this model, experimental data [2], and literature, there are still a few limitations to this model. First, data used to develop the model [2] were taken for an extraction time of 14 days, and a temperature range of 20 °C to 35 °C. Additionally, the adsorption works performed by Beaver [19] and Medina-Plaza [20] extend from 0 to 15% ethanol by volume solutions. Extensions of this model to extraction times greater than 14 days, or outside of the temperature and ethanol ranges, would necessarily be extrapolations. All kinetic parameters are assumed to be a function of temperature only; ethanol dependence is captured in the adsorption equilibrium relationships.

The proposed model groups all phenolics into three pseudo-species (anthocyanins, skin tannins, and seed tannins), rather than examining individual compounds. Further, anthocyanin concentrations were measured in Lerno [2] by Reversed Phase High Performance Liquid Chromatograph (RPHPLC) with a diode array detector. While the data indicate a decrease in free anthocyanin concentration towards the end of fermentation, the reaction products are not clear. Further improvements to this model would include parsing out the actions of specific phenolic to better predict overall anthocyanin and tannin behavior.

Finally, this model assumes a homogenous mixture of grape solids and liquid. This is a tolerable assumption in a small fermenter, which is frequently pumped-over, such as the partially full 120 L working volume fermenters used in Lerno [2] to collect the data used in this model. Diffusive-convective mass transfer will need to be considered when this work is extended to large industrial tanks.

Despite these limitations, this model successfully predicts experimental behavior over a wide range of processing temperatures and times, and is applicable to most red wine fermentations of industrial interest.

## 4. Material and Methods 

### 4.1. Grape Skin and Seed Microscopy

To further understand the release of phenolics from skins and seeds, we collected Cabernet Sauvignon skin and seed samples daily from duplicate 2000 L pilot fermenters using Yolo County fruit at the UC Davis Teaching and Research Winery during the 2018 harvest. Multiple seed and skin samples were pulled for analysis from the top and bottom of both fermenters.

Grape samples were rinsed with water, skins were removed from the berry, pulp was scraped off with a razor blade, skins were placed on a slide, hydrated with water, and sealed via cover slip with the outside of the skin facing up. Seeds were removed from the berry, rinsed with water and cleaned with a paper towel, sectioned via razor blade, placed on a slide, hydrated with water, and sealed via cover slip.

Bright Field and Epifluorescence microscopy were performed with a LEICA DM4000 B Microscope (Leica Microsystems, Wetzlar, Germany) with a Lumen Dynamic X-Cite 120 LED Box (Excelitas, Waltham, MA, USA). Spectrum Aqua (SAQ) and G/R (Green/Red) filter cubes were used to perform the epifluorescence microscopy, the SAQ filter cube having an excitation filter of D 436/20, a dichromatic mirror for 455 wavelength light, and a suppression filter for D 480/30, and the G/R filter cube having a BP (Band Pass) 490/20 and BP 575/30 excitation filter, a dichromatic mirror for 505 and 600 wavelength light, and a suppression filter for BP 525/20 and BP 635/40. No fluorophores were used to stain the samples. Images were taken with the LAS EZ software used for the LEICA DM4000 B. Length measurements were determined via measuring pixel lengths, with the length bar provided from the LEICA microscope as scale.

### 4.2. Calculations

Curve fittings were performed in Microsoft Excel using the built in regression fitting tools at 20 °C, 25 °C, 30 °C, and 35 °C on a Dell laptop running Microsoft Windows 10. Early time points were used to fit release kinetics (as concentration of free phenolics and reactant products is low), while late time points were used to fit reaction kinetics (as release has largely ceased). The derived equations were numerically integrated using COMSOL (Version 5.4, Stockholm, Sweden) Reaction Engineering module.

## 5. Conclusions

This work represents both an extremely useful predictive tool and a critical advancement to the understanding of the mechanisms underlying phenolic extraction during red wine fermentation, especially the extraction of seed tannins. This model can be used directly by winemakers to optimize extraction by manipulating temperature, fermentation rate, and press timing. The model could also be coupled to a diffusive mass transfer system for red wine fermentations, as developed by Miller [22,23] to account for spatial heterogeneity in commercial fermentations and predict spatial gradients in phenolic concentrations, akin to the experimental work in Lerno [4].

## Figures and Tables

**Table 1:** Activation Energies for Anthocyanin Degradation.

Work	Substrate	Activation Energy, kJ/mol
This work	Grapes (Cabernet Sauvignon)	107.3
Rhim [14]	Grapes (*Vitis Rotundifolia*)	92.8
Verbeyst [15]	Strawberries	94.4
Kechinski [16]	Blackberries	80.4
Wang [17]	Blackberries	59.0–75.5

**Table 2:** Arbitrary regression functions used to describe the temperature dependence of each parameter.

Parameter	Symbol (Unit)	Fit as a Function of T (°C)
Skin-Tannin Release Constant	k_G,Tan,Skin_ (1/h)	y = −0.000014T^2^ + 0.001554T − 0.019916
Skin-Anthocyanin Release Constant	k_G,Anth,Skin_ (1/h)	y = −0.0000081T^2^ + 0.0011173T − 0.0095045
Seed-Tannin Release Constant	k_G,Tan,Seed_ (mg/L/h)	y = 0.00089T^2^ − 0.00703T + 0.19030
Unavailable Anthocyanin Concentration	C_Anth,U_ (mg/L)	y = −0.53615T^2^ + 16.57825T + 75.70350
Unavailable Skin-Tannin Concentration	C_Tan,U_ (mg/L)	y = −25.1835T + 1080.7055
Anthocyanin Reaction Rate	k_R,Anth_ (mg/L/h)	See Arrhenius Parameters

**Table 3:** R^2^ (Goodness of Fit) for Extraction Model Fits at each temperature.

Temperature	Anthocyanin Fit R2	Tannin Fit R2
20 °C	0.93	0.99
25 °C	0.93	0.96
30 °C	0.84	0.99
35 °C	0.84	0.94
